# Task shifting from general practitioners to nurses in out-of-hours primary care: an explorative case study of team-based practices

**DOI:** 10.1080/02813432.2025.2490911

**Published:** 2025-04-15

**Authors:** Katrine Bjørnshave Bomholt, Anna Mygind, Mette Amalie Nebsbjerg, Morten Bondo Christensen, Linda Huibers, Viola Burau

**Affiliations:** a Research Unit for General Practice, Aarhus, Denmark; b Department of Public Health, Aarhus University, Aarhus, Denmark

**Keywords:** Primary health care, out-of-hours, task shifting, interprofessional collaboration, general practice, Team-based practice

## Abstract

**Background:**

Out-of-hours primary care (OOH-PC) is essential for treating urgent health problems. However, the high demand for these services has increased the workload.

**Objective:**

To investigate the interprofessional collaboration in OOH-PC and task shifting from general practitioners (GPs) to nurses, specifically the professional practices and the perceived experiences of GPs and nurses.

**Methods:**

This explorative case study was based on observations and interviews, using normalisation process theory as the theoretical framework. Observations were conducted in two OOH-PC clinics in the Central Denmark Region, followed by individual semi-structured interviews with five GPs and six nurses working in these clinics. Data were collected from March to October 2022.

**Results:**

GPs and nurses worked together in a team-based workflow based on different roles and skills but joint tasks. The nurses handled patients with injuries and performed diagnostic tests. A team-based workflow with task shifting was supported by familiarity within the team, with nurses performing informal coordination tasks and having formal support like protocols, training, and GP supervision. GPs and nurses appreciated the team-based workflow, as it facilitated efficient resource use and high job satisfaction. However, both groups expressed concerns about the sustainability of OOH-PC clinics.

**Conclusion:**

Task shifting from GPs to nurses in OOH-PC is feasible in a team-based workflow, resulting in efficient use of available resources and high job satisfaction. However, task shifting should not aim to replace GPs with nurses. Instead, their roles and skills should be seen as complementary, which calls for task sharing.

## KEY POINTS

- This study investigated team-based collaboration practices in out-of-hours primary care clinics, focusing on task shifting from general practitioners (GPs) to nurses.
- GPs and nurses worked together in a team-based workflow, where nurses independently handled most of the patients with injuries and performed diagnostic tests; this workflow resulted in efficient use of available resources and high job satisfaction.
- A team-based workflow with task shifting was supported by familiarity within the team, with nurses performing informal coordination tasks and receiving formal support in the shape of training, protocols, and accessible supervision from a GP.
- Task shifting should not aim to replace GPs with nurses. Instead, the roles of GPs and nurses should be seen as complementary, which calls for task sharing.

## Introduction

Out-of-hours primary care (OOH-PC) services are essential for providing timely, adequate care for urgent health problems with easy access outside regular working hours [1,2]. The demand for these services has increased due to an ageing population and rising multimorbidity, which places considerable pressure on healthcare systems [3–6]. In many Western countries, OOH-PC is coordinated by GP cooperatives, although the organisational models vary [1,7,8]. The shortage of general practitioners (GPs) has caused gaps in the provision of OOH-PC services and increased the workload for existing staff [9]. To address these challenges, new models of care, such as task shifting, have been proposed [1,3,10]. Task shifting involves delegating specific responsibilities from GPs to nurses and other healthcare professionals to optimise workforce usage and maintain high-quality patient care [11,12].

The WHO defines task shifting as delegating tasks to less specialised health professionals, thereby enabling all providers to practice within their full scope of expertise [13]. Several studies have indicated that task shifting from GPs to nurses is a promising and feasible healthcare model in daytime general practice. It has been shown to provide safe care and high patient satisfaction [11,12,14–16]. Despite strong evidence in favour of task shifting in daytime primary care, more research is needed on its application in OOH-PC settings. The evidence from daytime care cannot be applied directly to OOH-PC due to substantial differences between the two settings. In daytime general practice, the nurses primarily handle chronic diseases, whereas OOH-PC deals with patients with acute health issues [1]. Additionally, there is a level of familiarity among GPs and nurses in daytime general practice that does not exist in OOH-PC as GPs and nurses work in changing shifts [8,17]. The rotating team compositions may result in unfamiliarity among team members and affect their collaboration.

Task shifting in OOH-PC has mostly been restricted to telephone triage, which has proven safe and efficient [18–20]. In clinic consultations and home visits, task shifting is a relatively new but feasible concept [21–25]. Expanding the knowledge on the collaboration between GPs and nurses in OOH-PC is essential for improving collaborative practices and exploring the potential benefits of task shifting. Therefore, this study aimed to investigate the interprofessional collaboration in OOH-PC clinics and task shifting from GPs to nurses, specifically the professional practices and the perceived experiences of GPs and nurses.

## Methods

### Study design

We conducted an explorative case study based on observations and interviews, aiming to gain an in-depth understanding of the established professional practices in OOH-PC clinics and the GPs’ and nurses’ experiences with interprofessional collaboration and task shifting in these clinics. The study followed the COnsolidated criteria for REporting Qualitative research (COREQ) principles for reporting qualitative research (see Supplemental Material) [26].

### Theoretical framework

Normalisation process theory (NPT) was used as the theoretical framework [27]. NPT offers a valuable framework for understanding the mechanisms of introducing and integrating an innovation, e.g. interprofessional collaboration with task shifting from GPs to nurses, into everyday practice [28,29]. NPT has previously been used in primary healthcare research to examine how innovations become routine practice [30,31]. NPT identifies four constructs that act as key mechanisms for how people work to make innovations part of their everyday routines [28,29]. These constructs were operationalised to fit within the context of this study. *Coherence* revolves around how GPs and nurses perceive the meaning and purpose of interprofessional collaboration. *Cognitive participation* focuses on their active involvement and commitment, and how their skills and roles affect the collaboration. *Collective action* relates to their collaborative efforts and coordinated actions to enact and deliver the service. *Reflexive monitoring* refers to their evaluations and reflections on the impact of the collaboration between GPs and nurses.

### Setting

Denmark has tax-funded and free public healthcare for its residents [32]. General practice and OOH-PC are gatekeepers to secondary healthcare services. The study was conducted in the Central Denmark Region, where OOH-PC was provided by a large-scale GP cooperative at the time of data collection [5]. In case of acute health problems during OOH, patients can call the OOH-PC or emergency medical services at 1-1-2. Patients access the OOH-PC service through a single, region-specific telephone line. Danish GPs and GP trainees cover shifts in the regional OOH-PC service. Unlike the OOH-PC settings in many other countries, GPs perform telephone triage and determine the appropriate level of care: advice or treatment by telephone (with/without video use), referral to an OOH-PC clinic consultation, home visit by a GP, or referral directly to a hospital, e.g. emergency departments (EDs) in secondary care [33,34].

The Central Denmark Region has up to ten clinics varying in geograpy, size, staffing, facilities, opening hours, and level of collaboration with an ED. Most OOH-PC clinics provide care to patients in teams of GP(s) and a nurse, sometimes up to four GPs [29]. Team members differ in every shift. GPs are self-employed in their daytime practices but work under contract with the regional health services and are obligated to work OOH shifts. GPs are, on average, on call twice a month in either telephone triage, at a clinic, or conducting home visit. GPs are compensated on a fee-for-service model based on a remuneration coding system that also encompasses tasks performed by nurses. Before finalising a patient contact, the GP reviews the nurse’s notes to approve the treatment provided, as the GP has the overall responsibility for the consultation. Nurses working in OOH clinics are employed by the EDs and take rotating shifts between the ED and the OOH clinic. While the ED is formally responsible for managing the nurses, they are paid an hourly salary for their work in both settings. The nurses can complete additional training to independently manage patients with injuries, including the authority to take a medical history, conduct a physical examination, treat patients with, for example, bone traumas, wounds, eye and skin injuries, and refer to X-rays, but no authority to prescribe medication.

The OOH-PC clinics are equipped with some diagnostic testing devices, including blood glucose tests, pregnancy tests, rapid strep A tests, dipstick urinalyses, haemoglobin tests, and electrocardiograms. The clinics can refer to X-rays (located in the same building).

### Case selection

As an introduction to the project, the first author visited eight out of ten clinics in the Central Denmark Region from 4 July to 12 August 2021. Two clinics with long travel distances were not visited as they resembled clinics that had already been visited, for which we already had sufficient insights. The primary focus of the visits was interprofessional collaboration and task shifting. During these visits, observations and informal interviews were conducted with GPs and nurses working in the clinics. These visits provided valuable insights, as some clinics had already implemented task shifting for diagnostic tests and injury management. These clinics were identified as natural interventions for task shifting that was already happening in practice and subsequently became case studies for this study [35]. We invited two clinics with a high degree of task shifting because of their uniqueness [35]. Both clinics accepted our invitation to participate in an in-depth case study focusing on the established professional practices and the GPs’ and nurses’ experiences with interprofessional collaboration and task shifting in these clinics.

The clinics were similar in relation to context, team, nurse training, and level of interprofessional collaboration. Both clinics were co-located with an ‘acute clinic’ (no ED). The acute clinic acted as an ED satellite unit for trauma located away from the hospital. The ED was responsible for operating the acute clinic and was managed by the same nurse working with the GP in the OOH-PC clinic. The patients having an X-ray were transferred from the OOH-PC (primary sector) electronic system to the acute clinic (secondary sector). The nurse received telephone supervision from a doctor in the ED, both having access to the X-ray image, and the nurse could then complete the patient’s treatment in the acute clinic. The OOH-PC clinics were staffed by a team with one GP and one nurse working closely together. All nurses working in these clinics had additional training to manage patients with injuries independently. The nurses often had extensive experience in both hospital settings and the OOH-PC clinic.

However, some local differences existed between the two clinics. Clinic 1 was located at a small regional hospital with inpatient wards but without emergency admissions during OOH periods. The clinic was staffed with teams of one GP and one nurse who worked together from 4 pm to 11 pm The acute clinic remained open at night and was staffed by only a nurse. Clinic 2 was located at a regional health centre without inpatient wards but with outpatient clinics during daytime hours. The GP performed home visits during the shift spanning from 4 pm to 11 pm When the GP left the clinic for home visits, the nurse managed the OOH-PC clinic independently, receiving telephone supervision from the GP when needed. This clinic was closed during the night.

### Data collection

We conducted observations in the two OOH-PC clinics and performed individual semi-structured interviews with five GPs and six nurses working in these clinics (Table 1).

**Table 1.** Data collection and informants.

Clinic	Informant	Participated in data collection		Qualification as doctor or nurse (years)	Qualification as GP or nurse with additional training (years)	OOH-PC shifts during the past 3 months (n)
		Observations	Interviews			
1	GP1	X	X	+15	+10	2
	GP4	X	X	+20	+15	5
	GP5	X	X	+20	+10	9
	GP6	X		+30	+25	3
	N1	X	X	+25	+15	40
	N2		X	+15	0-5	5
	N4		X	+15	0-5	10
	N8	X		+10	0-5	28
	N9	X		+15	+10	50
2	GP2	X	X	+10	0-5	8
	GP3	X	X	+40	+35	14
	GP7	X		+20	+10	3
	GP8	X		+20	+10	1
	N3		X	+20	0-5	20
	N5	X	X	+30	+15	35
	N6		X	0-5	0-5	12
	N7	X		+40	+15	36
	N10	X		+25	0-5	31

GP: general practitioner; N: nurse; n: number.

Observations were performed from 16 March to 3 April 2022 by the first author and aimed to gain an in-depth understanding of the professional practices in the interprofessional collaboration between GPs and nurses. The observations were carried out over six days (approximately eight hours per day) on two weekdays and one Saturday/Sunday in each clinic, capturing fluctuations over weekdays and diversity in the staff involved. Eight GPs and six nurses participated during the six observation days (Table 1). All participants were unknown to the researchers before the study started. The first author conducted non-participant observations and followed the consultations by standing against the wall, where she had an unobstructed view of the interactions between patients and nurses/GPs. The first author attended all patient interactions after obtaining consent from the patients, none of whom declined participation. In a few consultations, informal interviews were conducted with the patients about their thoughts about being seen by a nurse. Informal interviews with the nurses were conducted between patient interactions. In cases where a nurse and a GP handled patients, the first author also observed the clinic consultations with the GP. Informal interviews with the GPs were also conducted between patient interactions. These observations provided valuable insights into how clinical practice was, particularly from the perspective of the nurses. As a result, interviews were conducted with the GPs first to get experiences and perspectives from their position, followed by interviews with the nurses. We used an observation guide inspired by NPT to focus our observations (see Supplemental Material), and field notes were taken during observations [27]. The volume of field notes taken during the observations was 13,005 words.

Subsequently, semi-structured interviews were conducted by the first author. Five GPs and six nurses from the participating clinics were interviewed in the period from 28 June to 26 October 2022. The aim was to gain an in-depth understanding of their interprofessional collaboration by exploring the perceived experiences of GPs and nurses. Purposive sampling was applied for recruitment to obtain variation in the general working experience and OOH-PC clinics. The six observed GPs who had regular shifts in the included clinics were invited to participate in interviews, and five agreed to participate. All observed nurses had several years of clinical experience and had taken numerous shifts in the OOH-PC clinic. The head nurses at the EDs, where the observed nurses were employed, assisted in selecting nurses for the interviews to ensure differing levels of experience in the OOH-PC clinic to achieve faithful representation. Six nurses were invited to participate, and they all agreed.

We used NPT and insights gained from the observations to develop an interview guide, with questions exploring each of the four NPT constructs (see Supplemental Material) [27]. The interviews took place at a time and place of the participant’s choosing (home; *n* = 3 GPs, work; *n* = 2 GPs and *n* = 6 nurses). All interviews were audio-recorded, and each lasted 39-75 min. A research assistant transcribed the interviews verbatim. The volume of transcribed interviews was 90,041 words.

### Data analysis

The first author read all observation notes and interview transcripts. First, a deductive approach was taken in the coding process using the NPT constructs. This ensured that all constructs were covered during data analysis and helped maintain a clear focus and structured analysis, preventing the study from becoming fragmented or overly broad. We did not open for other codes. Later, data was analysed using a more open approach according to Braun and Clarke’s thematic analysis strategy [36]. Hereby, codes in each NPT construct were analysed, and themes were identified. The initial themes were discussed and condensed into four main themes, one theme for each of the four NPT constructs but articulated to fit with everyday life in the clinic: ‘Working together in a team-based workflow’ (theme 1), ‘Supporting task shifting and team-based workflow’ (theme 2), ‘Cultivating task shifting and team-based workflow’ (theme 3), and ‘Maintaining sustainable task shifting’ (theme 4) (Table 2). This condensation process was guided by the study objectives and existing literature in the field, as this article aimed to contribute novel insights into the existing knowledge base. The findings were discussed in the cross-disciplinary research group with diverse professional backgrounds. KBB (first author) and MAN are PhD students and under specialist medical training for becoming GPs with clinical experience in urgent healthcare, emergency medicine, and general practice. MBC is an experienced GP and professor with profound research knowledge and clinical experience in urgent healthcare, general practice, and OOH-PC. LH is an associate professor and doctor with extensive OOH-PC and healthcare research knowledge. AM is an associate professor with extensive research experience in clinical behaviour, qualitative research, and intervention science. VB is a professor with extensive research experience and knowledge of the organisation of the healthcare system and change, healthcare professions, and health policy. To enhance rigour and trustworthiness, we continuously discussed the analytic process and reflected on our assumptions about the topic and interpretations of the analysis. As clinicians, the first author and other MDs in the research group could be solutions-oriented. We have witnessed the positive outcomes of interprofessional collaboration and task shifting from GPs to nurses, particularly in daytime general practice. Therefore, we approach this research with optimistic expectations about the potential benefits of task shifting. During discussions, AM and VB ensured that we remained open-minded and curious throughout the data collection and analysis. NVivo software was used to manage data and assist the coding process [37]. The full interviews were not translated into English. The first author translated the quotes cited in this article.

**Table 2.** NPT construct and themes.

NPT construct	Theme
*Collective action:* how the GPs and nurses collaborated and coordinated to enact and deliver the services.	Working together in a team-based workflow
*Cognitive participation:* how the GPs’ and nurses’ active involvement and commitment were expressed, and how their skills and roles affected the collaboration.	Supporting task shifting and team-based workflow
*Coherence:* how GPs and nurses perceived the meaning and purpose of the interprofessional collaboration.	Cultivating task shifting and team-based workflow
*Reflexive monitoring:* how the GPs and nurses evaluated and reflected on the impact of their collaboration.	Maintaining sustainable task shifting

## Results

The findings revealed four main themes, which will be elaborated below (Table 2).

## Working together in a team-based workflow

**Figure 1.**
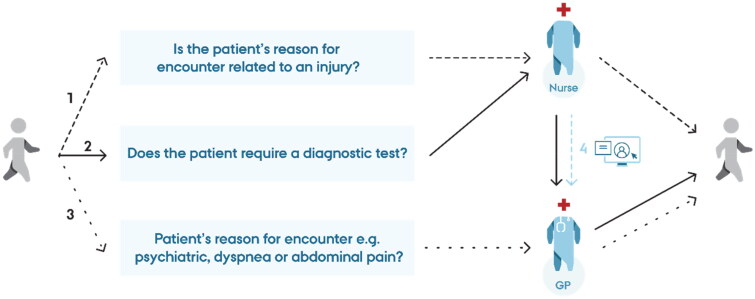
Patient pathway according to the reason for encounter. GP: General practitioner

The observations and the interviews showed that GPs and nurses worked closely together. In both clinics, the team-based workflow differed according to the individual patient’s reason for encounter (Figure 1).

The nurses explained that they obtained an overview of all patients referred to the clinic. The nurses triaged all patients with support from telephone triage notes in the medical records and observation upon arrival. Both GPs and nurses stressed that the GPs would always be responsible for treating patients with psychiatric conditions or complex health problems, such as digestive issues and respiratory difficulties (Figure 1, arrow 3). Our observations revealed that the nurses independently handled most patients with minor health problems, such as wounds, eye injuries, and bone traumas (Figure 1, arrow 1). However, each patient was assessed individually, and deviations from the usual task distribution were made if desirable. In a few cases, the nurse asked the GP for advice on treatment or prescription of medication, but most patients were handled independently by only one healthcare professional.

If the nurses found it relevant, they could independently refer the patient to an on-site X-ray and discuss images with an ED physician by phone. In most cases, the nurses could complete the treatment of patients at the clinic. Furthermore, the nurses assessed the need for diagnostic tests based on the telephone triage notes, communication with the patient, or a joint decision made with the GP. The nurses would then usually take the tests when the patient arrived (Figure 1, arrow 2). In a few cases, the GP performed a diagnostic test if the nurse was busy. A nurse described how the team-based workflow resulted in a joint responsibility in caring for patients:

"If there’s one [patient] I can take or prepare, then it feels more like ours. They aren’t yours [the GP’s] or mine. They are *our* patients. And then there are some that I can handle, but that’s just a help. It’s not like I think they’re only mine because maybe I think I can finish it, but then maybe I can’t [finish alone] after all. So, they [the patients] are ours. That’s how I see it." (N2)

Accordingly, a GP explained that the flow created by the nurses, e.g. performing diagnostic tests, allowed the GPs to proceed directly and promptly with medical history-taking, physical examination, diagnosis, and treatment. The distribution of roles and skills within the team-based workflow involved only little communication and seemed implicit for both GPs and nurses. Since the GPs were responsible for the treatment of all patients, they reviewed all the nurses’ medical records (Figure 1, arrow 4).

## Supporting task shifting and team-based workflow

### Familiarity and trust within the team

The nurses worked primarily in the OOH-PC clinics, even though they were formally employed at the ED. Consequently, they had many OOH-PC shifts during the week. In contrast, all GPs mainly worked in their daytime clinics and described themselves more as guests in the OOH-PC clinic, as they only occasionally had shifts there. The nurses’ numerous shifts meant that GPs frequently collaborated with the same few nurses despite the GPs’ few shifts in the clinic, which fostered familiarity and trust within the team. This familiarity and trust enabled GPs to confidently engage in the distribution of their roles and hand over some tasks to the nurses. One GP elaborated on the impact of knowing the nurses well:

“They operate very independently, the nurses […]. They are experienced. And I trust them because I’ve gotten to know them over time. So, it has become a very trusting collaboration that we have, where they can easily conduct their independent consultations, and I just read through the notes [which the nurses have written in the medical records]. I know and can trust that they have done a good job.” (GP2)

The nurses found that when GPs knew them well, the GPs were less sceptical and more supportive of the distribution of roles and tasks.

### Nurses performing informal coordination tasks

Having frequent OOH-PC shifts positioned the nurses as essential practical organisers in the OOH-PC clinic. This role encompassed various informal coordination tasks, such as managing IT tools, cleaning and replenishing equipment, and ensuring the availability of necessary resources. Consequently, the nurses developed deep insight into clinic equipment and operational processes. A nurse explained how this proactive approach was essential to maintaining an effective patient flow without prolonged waiting time and ensuring the efficient management of patient care:

“I definitely try to keep an eye on things. I really try to maintain a good flow because it’s important to me. If we don’t keep up, I know things will pile up too much, and we’ll have patients who end up waiting all around. So, I make a big effort, even though I handle my own independent treatment tasks, to also take care of things like checking blood sugar, measuring CRP, or taking a throat swab if I see it’s needed. I can do those things, including urine tests, so that the GP can stay in the consultation room, and the next patient will be fairly ready to go in.” (N1)

Some GPs highlighted that the overview made by nurses contributed to ensuring a streamlined workflow, comparing the level of efficiency with that in another OOH-PC clinic without close interprofessional collaboration and task shifting. One of them described working in the other clinic as follows:

“All that time I must spend walking back and forth in the hallway, taking a CRP, and waiting for a urine sample from someone. And there’s no collaboration […] People [patients] are still sitting there when you pass [the waiting room] for the seventh time to fetch something […]. It was so unsatisfactory.” (GP2)

Working in the other clinic with low level of interprofessional collaboration and task shifting is characterised by many frictions, which interrupt the workflows of GPs. This highlights the central role of nurses in ensuring informal coordination.

### Formal support like protocols, training, and supervision

The nurses could independently assess and treat most patients with injuries in the clinic because of additional training in specific health problems, such as wounds, eye injuries, and bone traumas, described in formal protocols. These protocols ensured that GPs were well informed about the tasks nurses could perform independently, which gave GPs confidence in the nurses’ capabilities. Conversely, the protocols served as clear guidelines for nurses, providing legal assurance for the tasks they could perform independently. Additionally, the nurses gained significant experience by frequently performing these tasks during their numerous shifts at the clinic, which helped them feel secure in these skills. The GPs highlighted that these nurses had become highly skilled in managing minor injuries, and, due to their extensive hands-on experience, the nurses were often better skilled than GPs at handling these health problems. A GP explained:

“As doctors, we’re moving further and further away from handling the kinds of tasks that they [the nurses] deal with. We also have a nurse [in the daytime], who takes care of all these injuries, or at least most of them. So, they’re much more experienced in doing this work than we [GPs] are. We can do it, of course, but honestly, they’re probably better at it than us because they do it all the time.” (GP2)

Furthermore, the nurses described their easy access to GP supervision as necessary for managing patients independently. Some health issues went beyond the protocols or fell within a grey area, where GP guidance enabled the nurse to complete patient care independently or quickly transfer the patient back to the GP. The nurses did not have the authority to prescribe medicine, and the GPs held the ultimate responsibility for the care provided by the nurses. Therefore, the GPs’ supervision was necessary.

## Cultivating task shifting and team-based workflow

### Efficient patient care

The GPs and nurses both experienced that they formed a well-functioning team with different roles and skills, but joined tasks. A GP elaborated on how their complementary roles and skills supported an effective, well-coordinated patient care process:

“It would be challenging to manage all patients within a reasonable timeframe if we could not distribute the workload between us. […] Things can suddenly take longer than expected, so it’s nice to have someone who can independently proceed with some of the tasks while I am occupied elsewhere. And the other way around. Of course, it [the team] could consist of two doctors, but I think that the roles are distributed in a suitable manner when it’s a GP and a nurse. Because then we don’t have to handle the same tasks.” (GP1)

GPs and nurses experienced that the shared understanding of their differing expertise allowed them to focus on their respective strengths. The GPs found their time well spent and their skills appropriately used, as they could concentrate on diagnostics and treatment and devote more time to patients with complex health issues. Likewise, the nurses found that their experience and special skills were well used; they could work independently, manage injuries, and apply their competencies in the collaborative setting.

### Improved job satisfaction

Both GPs and nurses expressed high job satisfaction, specifically because of the team-based workflow, the efficient use of resources, and the interprofessional relationship. A GP described a good shift like this:

“When I feel that we have done a great job, when I believe we have been effective, and I think we have helped some people in a really good way. And we’ve had a great time doing it. And then we’ve pulled it together by working as a team.” (GP2)

GPs and nurses explained that their high job satisfaction stemmed from a sense of functioning as a cohesive team instead of working parallel to each other. GPs also drew comparisons with other OOH-PC clinics and other settings characterised by less interprofessional collaboration, where they had worked previously. Here, they frequently experienced a lack of teamwork with nurses, which often caused feelings of isolation, helplessness, and frustration. These feelings were not confined to personal concerns; they also extended to their apprehensions about patient well-being and efficient allocation of healthcare resources.

## Maintaining sustainable task shifting

### Concerns about sustainability

Both nurses and GPs expressed concerns regarding the long-term sustainability of the team-based workflow and task shifting in OOH-PC clinics. These concerns were related to maintaining the existing practice within the context of the involved clinics and the feasibility of expanding similar practices to other OOH-PC clinics, where team-based practice and task shifting were either less established or absent. Concerns existed at multiple levels. At the individual level, GPs and nurses emphasised the need for professional acceptance of task shifting. The GPs also raised concerns regarding clinical responsibility, particularly when nurses took on expanded roles in patient diagnosis and treatment. On the other hand, some nurses expressed worry that task shifting could increase their workload and lead to a segmented approach to patient care, where responsibilities would be divided into "yours and mine" rather than fostering a fully integrated team-based approach. However, both groups agreed that the most significant challenge was recruiting nurses with the necessary qualifications and experience to operate effectively in OOH-PC clinics.

### Newcomers in a team-based practice with task shifting require extra attention

GPs and nurses indicated that the team-based workflow was disrupted whenever new GPs or nurses joined the team, which regularly happened in the OOH-PC clinics. New team members tended to unsettle the implicit understanding and shared acceptance of the established distribution of roles and tasks. GPs noted that new nurses were generally less familiar with the workflows and less experienced in their predefined tasks, e.g. injuries and diagnostic tests, thus requiring more formal delegation. Furthermore, GPs were concerned about task shifting, as they had the treatment responsibility for patients in the clinic and therefore needed to know the nurses well to feel safe about handing over tasks.

“I am much more at work when there is someone new, even though they are skilled and likely to be competent in their work. But I am much more at work because I have to get them started. They do not initiate things entirely on their own as the others do. And I end up doing more tasks and being more engaged because I do not know them, so I need to ensure that what they send out the door is okay.” (GP2)

Conversely, some nurses experienced that new GPs needed guidance to understand and support the existing distribution of roles, which was crucial for sustaining task shifting and redistributing responsibility.

### Differentiation between experience levels of nurses

A source of frustration, particularly among experienced nurses, was the use of uniform protocols that did not differentiate between experience levels. The formal protocols were aimed at the lowest common denominator without considering different experience levels among the staff. One experienced nurse commented:

“I sometimes feel that our competencies remain unrecognised, as the bar is set low for those of us who are more experienced to accommodate those who are less experienced, and more consideration is given to those who are less experienced, rather than allowing the experienced ones to lift the less experienced to a higher level.” (N1)

Consequently, the protocols were considered limiting, as they failed to recognise and fully utilise the nurses’ extensive experience. Still, new nurses sometimes felt uncomfortable when experienced colleagues deviated from these protocols, as they perceived these actions as an expansion beyond established practice.

## Discussion

### Statement of principal findings

The observations and interviews showed that GPs and nurses worked together in a team-based workflow based on different roles and skills but with joined tasks. The nurses handled patients with injuries and performed diagnostic tests, whereas the GPs handled complex diseases. A team-based workflow with task shifting was supported by familiarity within the team, nurses performing informal coordination tasks, and formal support like protocols, training, and GP supervision. GPs and nurses greatly appreciated the team-based workflow; they found that it provided efficient use of available resources and high job satisfaction. However, both groups expressed concerns about the sustainability of the interprofessional collaboration with task shifting in OOH-PC clinics.

### Strengths and limitations of the study

An essential strength of this study was the inclusion of both GPs and nurses who actively engaged in team-based practices, thereby providing a comprehensive view of the interprofessional collaboration in OOH-PC clinics. Another strength was the combined data from observations in collaborative practices and interviews with GPs and nurses. The observation aspect allowed us to identify the tacit and implicit behaviours that played a role in collaborative practices. Interviewing both types of health professionals permitted the exploration of interview topics from different viewpoints, thus enhancing the credibility of the study. Moreover, the study was conducted by an interdisciplinary team of researchers, including medical doctors and GPs (KBB, MAN, MBC, and LH), quantitative researchers (KBB, MAN, MBC, and LH), and experienced qualitative researchers (AM, VB). To ensure trustworthiness, we continuously discussed our choices in the cross-disciplinary research team, which raised the awareness of potential bias and preconceived assumptions.

However, the study also had limitations. First, the study was conducted in Danish OOH-PC clinics, where OOH-PC is provided by a large-scale GP cooperative. The teams in the two clinics were small, with only one GP and one nurse, and all observed nurses had several years of clinical experience and had taken numerous shifts in the OOH-PC clinic. Our findings may not be directly transferable to OOH-PC clinics with larger teams and/or with nurses without additional training and little experience in the OOH-PC setting or other healthcare systems, as healthcare systems and organisations can vary within and between countries. However, the clinics were precisely selected because they stood out from the rest. Second, we included clinics with the highest level of task shifting. The data collection captured practices within a specific context and timeframe, which might not represent broader patterns. Clinics with low levels of task shifting could have been included to get diverse perspectives and experiences in the data and provide a better understanding of the barriers to task shifting in OOH-PC clinics. Moreover, although the first author’s familiarity with clinical practice added depth to the analysis, it might have introduced subtle bias in favour of the GP perspective. Being solutions-oriented clinicians, the first author and other MDs in the research group could have been motivated to identify solutions to address broader challenges. Still, during their practice as clinicians, they have witnessed the positive outcomes of interprofessional collaboration and have been taught to optimise workflows. This might have introduced subtle bias in favour of the positive aspect of task shifting. However, efforts were made to mitigate potential bias through group discussions. We continuously discussed our choices in the cross-disciplinary research team to raise our awareness of potential bias and preconceived assumptions. Third, the first author was inexperienced in conducting observations and interviews. This could have limited the diversity of perspectives and experiences in the data. Further training or pilot interviews could have strengthened the process. However, group discussions ensured that a wide range of perspectives were presented.

### Findings compared to other studies

A review has shown that nurses can provide quality of care comparable to that of GPs for various types of patients in OOH-PC, although they see mainly patients presenting with less urgent and less complex health problems [38], as also found in the present study. However, little evidence exists on the professional practices and perceived experiences of GPs and nurses in OOH-PC clinics with task shifting from GPs to nurses. We found that nurses managed most patients with injuries and performed diagnostic tests independently, which aligns with the findings of previous studies on the potential of new roles and skills for nurses in OOH-PC [39,40]. GPs and nurses identified skills such as suturing wounds, referring to X-rays, and treating lower urinary tract infections [40]. Both GPs and nurses experienced that the interprofessional setup positively affected resource utilisation, which has previously been reported in existing literature on interdisciplinary collaboration [30,31]. Although diagnostic testing in OOH-PC is relatively rare, direct access to diagnostic facilities has been suggested to optimise efficiency in these settings [41]. However, evidence seems to indicate that GPs performing more point-of-care testing tend to prescribe more antibiotics compared to GPs performing fewer tests, which could raise concerns about the implications of increased diagnostic access on prescribing practices [42].

Nurse education through formal protocols and access to GP supervision was key to enabling GPs and nurses to actively participate in interprofessional collaboration with task shifting in OOH-PC clinics. Protocols clarify roles, reduce inefficiencies, and foster trust, particularly for less experienced staff [43]. In addition, confidence should be built up through appropriate training, support, and mentoring [43]. Individualised role-specific training, particularly for less experienced staff, may build confidence, minimise stress, and integrate other health professionals into the professional team [44]. Specific training in OOH-PC contexts, such as managing acute conditions and performing diagnostic tests, has been found to improve team efficiency and alleviate GP workload [45]. Regular clinical mentorship and access to a designated GP for guidance have been shown to promote confidence, job satisfaction, and autonomous practice, while also providing reassurance and validation of clinical decisions [24,46]. Continuous professional development and stakeholder involvement in training design is essential to align systems with organisational goals and enhance scalability [45,47].

However, the primary challenge lies in developing a successful model for interprofessional collaboration with task shifting in OOH-PC clinics [31,48]. Support from professional associations is essential to successfully implement new tasks for nurses in OOH-PC, role standardisations, and long-term political planning [49]. Additionally, acceptance of the nurse’s role among health professionals and the public is necessary [45,50]. A study exploring whether nurses could perform new tasks in an OOH-PC clinic found that the GPs were sceptical, while the registered nurses and patients were generally supportive [40]. Our study supports the previous evidence that GPs and nurses should work in teams characterised by complementary skills and mutual support [48,51]. Additionally, continuity in staffing, where the same nurses frequently collaborate with the same GPs, has previously been shown to foster familiarity and trust within the team, which is crucial for mutual support [17]. We found that informal coordination, predominantly managed by nurses, ensured a good patient flow, which further supported the team-based workflow. Likewise, previous research has demonstrated that informal coordination often complements formal processes to enhance collaboration [52]. Addressing this dynamic is essential for maintaining the positive outcomes of interprofessional collaboration with task shifting. It is also essential for ensuring the continuous delivery of high-quality care in OOH-PC settings.

### Implications in practice and future research

This study highlights key elements in the successful implementation of well-structured team-based workflows and task shifting in OOH-PC clinics. To achieve sustainable task shifting, responsibility allocation is important to facilitate optimal resource utilisation and better patient outcomes. Thus, it is crucial to implement structured role definitions in OOH-PC to enable both GPs and nurses to practice within their full scope of expertise. Formal protocols, training, and GP supervision enabled nurses to effectively expand their roles. Consistent training programmes and clear protocols are essential to maintaining high standards of care and successful task shifting. However, these protocols should be differentiated according to the experience of each nurse. Furthermore, familiarity and informal coordination within teams, often led by nurses, were critical for effective collaboration. Therefore, continuity in staffing is essential for building trust and improving team performance. Yet, concerns exist about how to ensure sustained task-shifting models, especially maintaining a trained workforce. This indicates a need for long-term strategies to ensure sustainable and expandable task-shifting models in OOH-PC.

Future research should explore the long-term sustainability of task-shifting models in OOH-PC settings, with particular attention to factors that support or hinder maintaining a well-trained workforce and clearly defined roles. Additionally, understanding the perceptions of patients and healthcare professionals regarding expanded nurse roles is crucial, as public and professional acceptance is key to successful implementation. Research should also examine structural factors influencing task shifting, such as how organisational frameworks, policies, and systemic conditions facilitate or hinder effective practices. While NPT provided a valuable framework for understanding the implementation and integration of task shifting in OOH-PC clinics, alternative theoretical approaches could address specific challenges in other clinics. The Consolidated Framework for Implementation Research (CFIR) is one such approach that is particularly well-suited for identifying and addressing barriers and facilitators to implementation across diverse settings [53]. CFIR offers a more comprehensive perspective on organisational change by examining organisational culture, leadership engagement, and external policies. For clinics with low levels of interprofessional collaboration or limited task-shifting practices, the adaptability of CFIR allows interventions to be tailored to specific needs and contexts, thereby enhancing scalability and sustainability. Combining insights from NPT and CFIR could enable more targeted strategies to address implementation challenges, promote effective team-based practices, and ensure the long-term success of task-shifting initiatives.

## Conclusion

Task shifting from GPs to nurses in OOH-PC is feasible in a team-based workflow; it provides efficient use of available resources and high job satisfaction among healthcare staff. Task shifting should not aim to replace GPs with nurses. Instead, the roles and skills of these two types of healthcare professionals should be seen as complementary, which calls for task sharing.

## Supplementary Material

Supplemental Material

## Abbreviations

GDPR: General Data Protection Regulation
GP: general practitioner
N: nurse
NPT: normalisation process theory
OOH-PC: out-of-hours primary care
n: number
