# Supplementary material for: Task shifting from general practitioners to nurses in out-of-hours primary care: an explorative case study of team-based practices
Source: Scand J Prim Health Care. 2025 Apr 15;43(3):626–38. doi: 10.1080/02813432.2025.2490911 (PMC12377107; doi:10.1080/02813432.2025.2490911)
Supplement: Supplemental Material [file IPRI_A_2490911_SM1322.docx]

COREQ (COnsolidated criteria for REporting Qualitative research) checklist

| **Topic** | **Item no.** | **Questions** | **Reported on**  **page no.** |
| --- | --- | --- | --- |
| **Domain 1: Research team and reﬂexivity** | | | |
| *Personal characteristics* | | | |
| Interviewer/facilitator | 1 | Which author(s) conducted the interview or focus group? | 9 |
| Credentials | 2 | What were the researcher’s credentials? e.g. PhD, MD | 11 |
| Occupation | 3 | What was their occupation at the time of the study? | 11 |
| Gender | 4 | Was the researcher male or female? | 1 |
| Experience and training | 5 | What experience or training did the researcher have? | 22 |
| *Relationship with participants* | | | |
| Relationship established | 6 | Was a relationship established prior to study commencement? | 9 |
| Participant’s knowledge of  the interviewer | 7 | What did the participants know about the researcher? e.g. personal  goals, reasons for doing the research |  |
|  |  |  | 9 |
|  |  |  |  |
| Interviewer characteristics | 8 | What characteristics were reported about the interviewer/facilitator?  e.g. bias, assumptions, reasons and interests in the research topic |  |
|  |  |  | 11-12 |
|  |  |  |  |
| **Domain 2: Study design** | | | |
| *Theoretical framework* | | | |
| Methodological orientation and theory | 9 | What methodological orientation was stated to underpin the study? e.g. grounded theory, discourse analysis, ethnography, phenomenology,  content analysis |  |
|  |  |  | 5-6 |
|  |  |  |  |
| *Participant selection* | | | |
| Sampling | 10 | How were participants selected? e.g. purposive, convenience,  consecutive, snowball |  |
|  |  |  | 7-10 |
|  |  |  |  |
| Methodological approach | 11 | How were participants approached? e.g. face-to-face, telephone, mail,  email |  |
|  |  |  | 9-10 |
|  |  |  |  |
| Sample size | 12 | How many participants were included in the study? | 9 |
| Non-participation | 13 | How many people refused to participate or dropped out? Reasons? | 9-10 |
| *Setting* | | | |
| Setting of data collection | 14 | Where was the data collected? e.g. home, clinic, workplace | 9-10 |
| Presence of non-  participants | 15 | Was anyone else present besides the participants and researcher(s)? |  |
|  |  |  | 9-10 |
|  |  |  |  |
| Description of sample | 16 | What are the important characteristics of the sample? e.g. demographic  data, date |  |
|  |  |  | Table 1 |
|  |  |  |  |
| *Data collection* | | | |
| Interview guide | 17 | Were questions, prompts and guides provided by the authors? Were these components pilot-tested? | 9-10 |
|  |  |  |  |
| Repeat interviews | 18 | Were repeat interviews carried out? If yes: How many? | No |
| Audio/visual recording | 19 | Was audio or visual recording used to collect the data? | 9-10 |
| Field notes | 20 | Were ﬁeld notes made during and/or after the interview or focus group? | 9-10 |
| Duration | 21 | What was the duration of the interviews or focus group? | 9-10 |
| Data saturation | 22 | Was data saturation discussed? | 11 |
| Transcripts returned | 23 | Were transcripts returned to participants for comments and/or | No |

| **Topic** | **Item no.** | **Questions** | **Reported on**  **page no.** |
| --- | --- | --- | --- |
|  |  | corrections? |  |
| **Domain 3: Analysis and ﬁndings** | | | |
| *Data analysis* | | | |
| Number of data coders | 24 | How many data coders coded the data? | 11-12 |
| Description of coding  tree | 25 | Did the authors provide a description of the coding tree? |  |
|  |  |  | No |
|  |  |  |  |
| Derivation of themes | 26 | Were themes identiﬁed in advance or derived from the data? | 11-12 |
| Software | 27 | What software (if applicable) was used to manage the data? | 11-12 |
| Participant checking | 28 | Did participants provide feedback on the ﬁndings? | No |
| *Reporting* | | | |
| Quotations presented | 29 | Were participant quotations presented to illustrate the themes/ﬁndings?  Was each quotation identiﬁed? e.g. participant number | 11-12 |
|  |  |  |  |
|  |  |  |  |
| Data and ﬁndings consistent | 30 | Was there consistency between the data presented and the ﬁndings? | 11-12 |
| Clarity of major themes | 31 | Were major themes clearly presented in the ﬁndings? | 11-12 |
| Clarity of minor themes | 32 | Is there a description of diverse cases or discussion of minor themes? | 11-12 |

**Observation guide**

| Coherence | Differentiation | How do GPs and nurses collaborate on division of tasks, decision-making and responsibilities? |
| --- | --- | --- |
|  | Communal specification | Which shared goals do GPs and nurses have for the interprofessional collaboration?  Which common advantages/disadvantages do GPs and nurses experience about the interprofessional collaboration? |
|  | Individual specification | How does the interprofessional collaboration affect the GPs’ and the nurses’ way of working? |
|  | Internalisation | Which components of the interprofessional collaboration are perceived as valuable and important?  Which components of the interprofessional collaboration are perceived as challenging? |
| Cognitive participation | Initiation | Are there key persons who drive the interprofessional collaboration and make others take part – and who are they? |
|  | Legitimation | To which extent do the GPs and nurses perceive the interprofessional collaboration as a legitimate part of their professional role? |
|  | Enrolment | Is there anything that motivates/demotivates GPs and nurses in relation to the interprofessional collaboration? |
|  | Activation | How do GPs and nurses underpin the interprofessional collaboration? |
| Collective actions | Interactional workability | How do GPs and nurses collaborate in out-of-hours consultations?   - How are formal documents/guidelines brought into play? - Which informal practices are applied? |
|  | Relational integration | How does the interprofessional collaboration affect the relations between GPs and nurses? |
|  | Skill set workability | Do GPs and nurses have the right skills for the interprofessional collaboration?  How do GPs and nurses obtain the right skills? |
|  | Contextual integration | Are there resources available to support the interprofessional collaboration?  How does the management support the interprofessional collaboration? |
| Reflexive monitoring | Systemisation | Which knowledge exists on the effects of the interprofessional collaboration? |
|  | Communal appraisal | Do GPs and nurses agree that the interprofessional collaboration is meaningful or not? |
|  | Individual appraisal | Which effects (positive/negative) do GPs and nurses assess the interprofessional collaboration to have on their professional role? |
|  | Reconfiguration | How do GPs and nurses optimise the interprofessional collaboration? |

**Interview guide**

| Collective actions | Would you try to describe the collaboration between you and the nurse/GP in the consultation in the out-of-hours medical service? e.g. who does what?   - Formal/informal practices - Other out-of-hours consultations - Different nurses/GPs - Division of labour, responsibility, communication - Over time   How do you assess your own and the nurses’/GPs’ skill set for working this way?   - In relation to different nurses/GPs   How do you perceive the support from the management for this way of working?   - Over time - Other consultations - Financial and political support |
| --- | --- |
| Cognitive participation | How do you perceive yours and the nurses’/GPs’ professional role in the collaboration compared with other units in the out-of-hours-service or previously?   - What factors do you believe to have driven the development and maintained the collaboration? (e.g. in case of new employees) - Are there any conditions that help motivate or demotivate the collaboration? |
| Reflexive monitoring | Which knowledge does your team have on how the collaboration works in the consultation in the out-of-hours service?   - Formal and informal? - Complaints - Evaluation, quality assurance - Staff meetings, meetings, project day - Informal: coffee chats   How do you perceive the existing extent of evaluation?   - Present or not? - Need/miss it? |
| Coherence | To which extent does your collaboration make sense for you as a GP/nurse?   - In relation to professional competence - In relation to task/responsibility (patient type, group, ”easy/difficult” patients) - Compared to other out-of-hours consultations - Compared to nurses/GPs with another background/competency |
